# Episodic rolling and transient attachments create diversity in sperm swimming behavior

**DOI:** 10.1186/s12915-014-0067-3

**Published:** 2014-08-16

**Authors:** Donner F Babcock, Petra M Wandernoth, Gunther Wennemuth

**Affiliations:** Department of Physiology and Biophysics, University of Washington, Seattle, WA 98195-7290 USA; Institute for Anatomy, University Hospital, Duisburg-Essen University, Essen, 45141 Germany

**Keywords:** Capacitation, CASA, Hyperactivation, Sperm competition, Sperm cooperation, Sperm motility

## Abstract

**Background:**

Frequency and asymmetry of the flagellar waveform of sperm are controlled by cAMP-mediated and Ca^2+^-dependent signaling pathways, but additional mechanisms modulate sperm swimming behavior. Here, high-speed imaging of free-swimming mouse sperm simultaneously reports flagellar waveform, orientation of sperm head, and swimming paths.

**Results:**

We found many sperm roll (rotate around their long axis) at intervals closely tied to flagellar beat frequency, allowing an asymmetrical flagellar beat to form linear averaged swimming trajectories. For non-rolling sperm, flagellar waveform asymmetry dictated circular path trajectories. Sparse rolling produced abrupt changes in swimming trajectories that occurred spontaneously, unaffected by blockade or engagement of cAMP- or Ca^2+^-mediated flagellar responses. Still other sperm loosely attached (tethered) to surfaces or other cells. Sperm tethered to each other in duos or trios could have narrowed swimming paths, allowing enhanced progression.

**Conclusions:**

We propose that transient episodes of rolling and reversible attachments are organizing principles that determine diverse swimming behaviors, which may have roles in selection of the fertilizing sperm.

**Electronic supplementary material:**

The online version of this article (doi:10.1186/s12915-014-0067-3) contains supplementary material, which is available to authorized users.

## Background

Mature mammalian sperm and eggs are produced and released in vastly disproportionate numbers. That disparity is nearly eliminated in the period between mating and fertilization, which prepares and selects the single (or few) fertilizing sperm from the many in the deposited semen [1]. For example, a recent study in mouse reported 9 ± 1 sperm in the ampulla 4 to 5 h after coitus [2]. Much attention has focused on possible roles of chemotaxis [3], thermotaxis [4], and rheotaxis [2] in guiding progress of those few sperm along the female upper reproductive tract to reach the egg. However, the question of how those few sperm are selected has received little attention.

In past work, we used stop-motion imaging to monitor the flagellar waveform of individual mouse [5–14] and human [15] sperm loosely attached (tethered) to the chamber surface, as we applied and removed various test substances by local perfusion. Those studies revealed several major features of the cAMP- and Ca^2+^-mediated signaling pathways that control the speed and symmetry of the flagellar beat.

In other laboratories, most work on free-swimming sperm has used commercial computer-assisted sperm analysis systems as described by Mortimer [16,17] to track swimming paths without imaging the sperm flagellum or recording the orientation of the sperm head. Here, we use a high-speed imaging approach to record images of both head and flagellum for subsequent kinetic and waveform analysis. The results provide new insights into how sperm swimming trajectories are determined, and show how transient attachments and reorientation produce various sperm swimming behaviors that may allow them to circumvent barriers to their progress, and thus have an active role in selection for fertilization.

## Results

Ideally sperm swimming behavior should be monitored simultaneously in x, y, and z planes. We simplified the task by use of shallow (20 μm) chambers and a 10× microscope objective with a similar (approximately 14 μm) depth of field. This combination provided video records of focused images that reported x and y locations for the entire cell, regardless of position in the z plane. Spatial and temporal resolutions (about 1 μm and 3 ms) were high. The recorded flagellar waveforms and swimming paths were two-dimensional projections of the real three-dimensional waveforms and trajectories. However, they provided new insights into the relationship between swimming behavior and the orientation of the plane of the predominantly planar flagellar beat.

### Complexity underlies linear mean swimming trajectories

**Figure 1:**
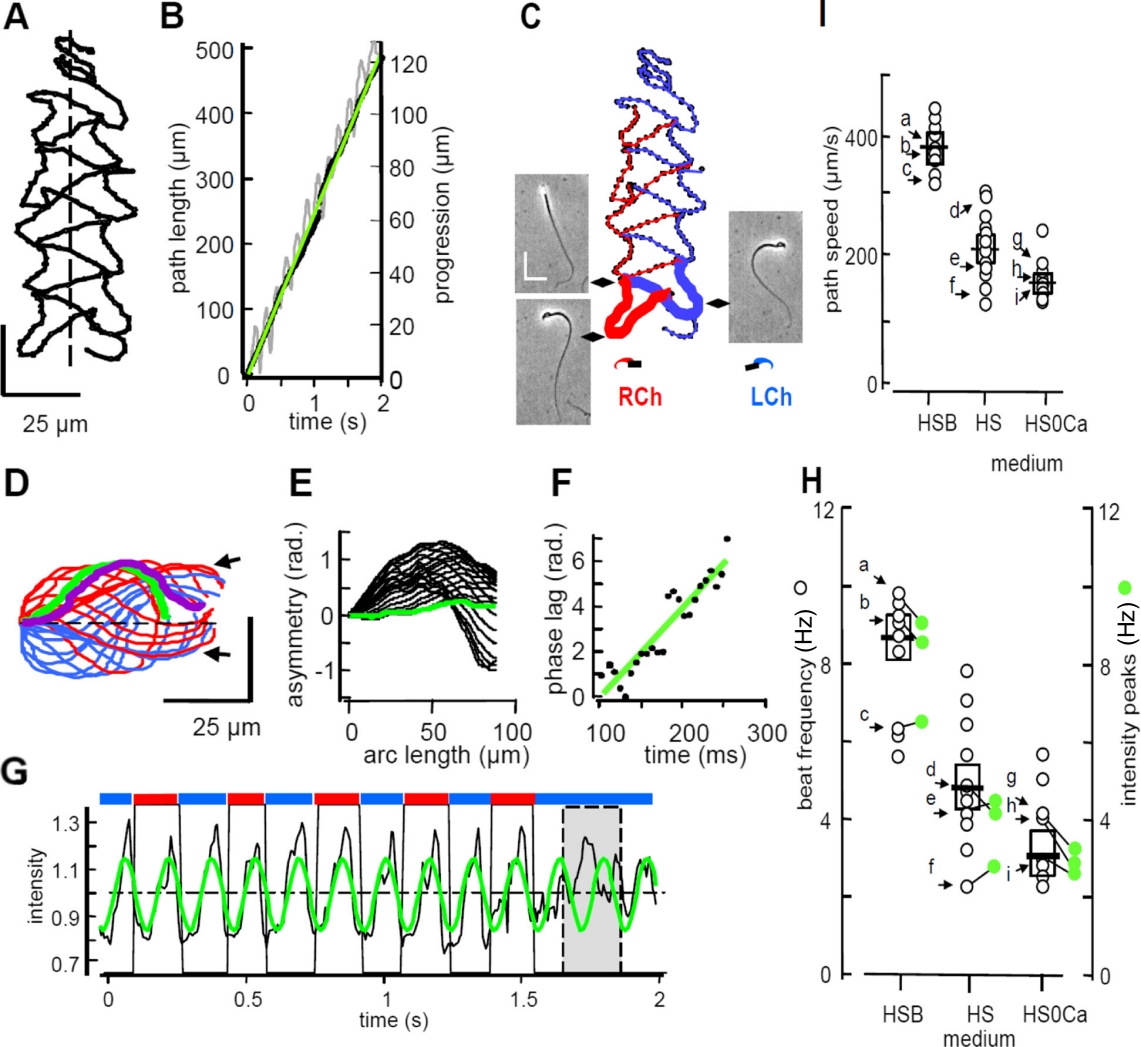
**Linear mean swimming trajectories comprise regularly alternating changes in sperm orientation synchronized with the flagellar beat cycle. (A)** Tracking a typical free-swimming sperm. Duration 2 s, sampling 150 frames per s (fps), medium HSB. Dashed line approximates mean swimming trajectory. **(B)** Time course of progress along swimming path. Point-to-point movement of head centroid defines path (black). Best fit linear regression lines (green) indicate mean path speed and progression (gray, linear distance from initial position). **(C)** Two initial segments of swimming track (heavy lines), flanking cell images at indicated track locations. Red, blue indicate cell orientation with right cheek (RCh), left cheek (LCh) facing bottom surface. Scale bar 25 μm. **(D)** Aligned flagellar traces (75 fps sampling) during initial RCh (red) and LCh (blue) segments. Initial trace green; final trace purple. Arrows indicate linear waveforms observed near RCh-to-LCh transition. **(E)** Flagellar waveform asymmetry from running averages of tangent angles (shear angles, in radians) for traces in D. Final, time-averaged shear angle is green. **(F)** Flagellar beat frequency (6.31 Hz) reported by phase lag of sine curves fitted to traces in D. Phase changes by 2π radians each beat cycle. **(G)** Normalized intensity from 7 × 7 pixel region-of-interest (ROI) enclosing the centroid (black) oscillates regularly (green). Red and blue bars (top) mark RCh, LCh orientation. Dashed box encloses interval when another motile cell interferes with intensity signal. **(H)** Flagellar beat frequency (black) correlates with frequency of ROI intensity peaks (green). Lines connect frequency pairs from same cell. Arrows and letters (a to i) indicate cells also analyzed as follows. **(I)** Path speeds for cells in resting medium (HS, n = 12 cells), activating HCO^3−^-fortified medium (HSB, n = 13 cells), or Ca^2+^-deficient media (HS0Ca, n = 12 cells). Note rank order within each medium is the same as in panel H.

Imaging of fields of mouse sperm in shallow chambers allowed manual tracking of the path of the centroid of the head of individual sperm. In a representative 2 s record (Figure 1A, Additional file 1), the averaged swimming path was linear, but showed alternating lateral excursions that were skewed to produce interwoven episodes of forward and retrograde progression. Although movement along this path occurred at a nearly constant speed (243 ± 1 μm s^−1^, Figure 1B), mean forward progression was episodic and slower (63 ± 0.5 μm s^−1^).

In Figure 1C, visual examination of the sequential images in this 2 s series indicated periodic reorientation of the plane of the falciform head of the mouse sperm, relative to the observer (insets), such that either the right or left ‘cheek’ was closest to the bottom of the chamber. We abbreviate these alternative orientations as RCh and LCh. Importantly, the transitions between orientations correlated with changes in lateral path trajectories. At this transition, the orientation of the head became briefly indeterminate. We examined the images of the initial, adjacent, paired RCh and LCh sequences (heavy lines) in more detail.

Manual tracing of the flagellum (Figure 1D) produced curvilinear waveforms for most of both sequences. However, groups of apparently linear waveforms were found during the transition between the RCh and LCh orientations, when the flagellar wave rotated into the z-plane as the plane of the head became briefly perpendicular to the chamber surface. Such tracings allowed determination of the time-averaged bending of flagellum (shear analysis), providing a measure of the asymmetry of the flagellar wave [7]. For the trace sequence of Figure 1D, the time-averaged bending produced a near-zero asymmetry for the proximal approximately 60 μm of the flagellum (Figure 1E). This low-asymmetry of the combined LCh and RCh sequence pairs is consistent with the nearly linear mean swimming trajectory shown in Figure 1A.

For the flagellar traces of the RCh and LCh sequences of Figure 1D, fitted sine curves had phases that increased over the 150 ms time course (Figure 1F). The slope of the best-fit regression line was 19.5 ± 1.6 radians s^−1^, indicating that the sinusoidal waveform cycled at 3.1 ± 0.3 Hz. The flagellar beat frequency (6.2 ± 0.6 Hz) was twice this value because of the phase shift produced by the RCh-to-LCh transition.

In the three images of Figure 1C, light scattered or reflected from the sperm head was brightest during the RCh-to-LCh transition. Figure 1G shows that similar changes in intensity occurred periodically over the entire 2 s image sequence. The normalized intensity oscillated by approximately 20% above and below the mean, peaking at, or slightly before, each change in orientation. Similar cycle frequencies were reported by best-fit analysis (6.31 ± 0.04 s^−1^) and by the mean intensity peak interval (6.36 ± 0.02 s^−1^). In this experiment, the frequency of intensity-peaks agreed closely with the frequency of the flagellar beat.

The frequency of the flagellar beat can be varied by adjusting the ionic composition of the medium [7,10]. Here, the 8.7 ± 0.5 Hz mean beat frequency in the bicarbonate-fortified (activating) medium HSB was nearly two-fold that in unfortified medium HS and nearly three-fold that in the Ca^2+^-deficient medium HS0Ca (Figure 1H). When the image sequences for representative individual cells a to i were also examined for variations in reflected light, the frequency of intensity peaks were in each case similar to the flagellar beat frequency determined as in Figure 1F, or by a Fast Fourier Transform method [12]. The swimming path speed for cells, including these same representative cells (Figure 1I), also depended on media composition. Not surprisingly, the path speeds for cells a to i followed the same rank order as that for beat frequency.

We conclude that linear swimming trajectories are composed of alternating episodes of laterally biased swimming, each of which has a non-progressive followed by a progressive component. We further found that the transition between these biased episodes was synchronized with the flagellar beat, and is reported by a transient increase in light scattered from the sperm head.

### Rolling produces the periodic changes in orientation for resting, activated, and hyperactivated sperm

**Figure 2:**
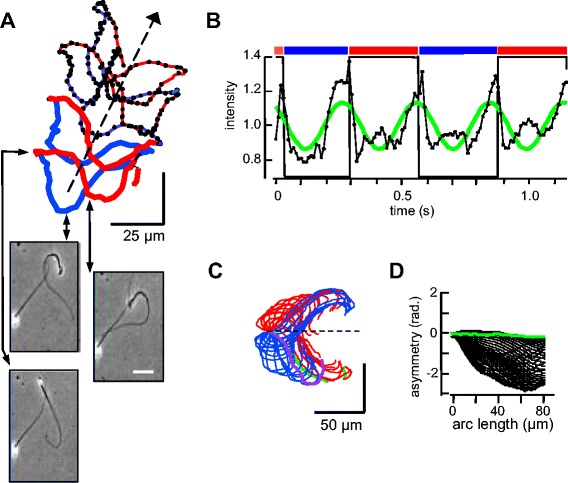
**Rolling produces regularly periodic changes in orientation for hyperactivated sperm. (A)** Tracking the head centroid of a free-swimming hyperactivated sperm. Duration 2 s, sampling 100 frames per second (fps), medium HS. Heavy red and blue lines indicate right cheek (RCh) or left cheek (LCh) orientation during the initial 1 s. Arrows indicate location of the stop-motion images below. **(B)** Intensity of light from a 7 × 7 pixel region of interest enclosing the centroid (black) also follows a sinusoidal path (green) that peaks near transitions between RCh and LCh orientations. **(C)** Aligned waveform traces sampled at 50 fps during the initial approximately 0.5 s of tracking. Initial trace is green; final trace is purple. Red and blue indicate cell orientation. **(D)** Flagellar asymmetry reported by the time-averaged tangent angle (shear angle).

The sequence that prepares sperm for fertilization includes two changes in motility. First, an early HCO_3_^−^-evoked activation of motility increases flagellar beat frequency of ‘resting’ sperm taken from the epididymis and vas deferens prior to exposure to male and female reproductive fluids. Second, a subsequent delayed ‘hyperactivation’ produces a transition from a low-amplitude, symmetric to a high-amplitude, asymmetric flagellar waveform [1,6,18,19]. Figure 2A tracks 2 s of the swimming path of a representative hyperactivated sperm. Its averaged trajectory also was approximately linear, with alternating lateral excursions that correlated with changes in LCh or RCh orientation. The inset images show the characteristic extreme curvature of the proximal flagellum midway in the LCh and RCh segments, and the linear waveform and bright head associated with the LCh-to-RCh transition. The mean path speed (135 ± 26 μm s^−1^) and forward progression (27 ± 2 μm s^−1^) of hyperactivated sperm (n = 7 cells) was slower, but on average the 23 ± 2% mean efficiency ratio (progression speed/path speed) was similar to that for cells examined prior to hyperactivation in medium HSB (23 ± 2%, n = 12) or medium HS (27 ± 2%, n = 8).

Hyperactivation does not disrupt the synchronization of the oscillating changes in intensity of light from the head with the RCh-to-LCh transition (Figure 2B). As for sperm examined prior to hyperactivation (Figure 1G), peak intensities occurred at, or slightly before, the transitions in orientation. Here, the frequency of intensity peaks was 3.7 ± 0.1 Hz. Because the oscillations in intensity are synchronized with the beat, they also report the flagellar beat frequency of hyperactivated sperm.

Aligned flagellar traces (Figure 2C) taken from the initial pair of complete LCh and RCh segments show the extreme flagellar curvature of hyperactivated sperm that also is seen in the images of Figure 2A. Figure 2D shows that together the similar flagellar waveforms observed during the LCh and RCh orientations produced a near-zero, time-averaged bending (asymmetry), consistent with the observed linear, averaged swimming trajectory.

In summary, Figure 2A-D reports that alternating RCh and LCh orientations produce near-linear trajectories for hyperactivated sperm, despite the extreme curvature and large amplitude of their flagellar waveform. These linear trajectories and the coordination of rolling with the flagellar beat cycle are maintained despite our use of chambers whose depth (20 μm) presumably restricts the waveform when the flagellum beats in the z-plane.

**Figure 3:**
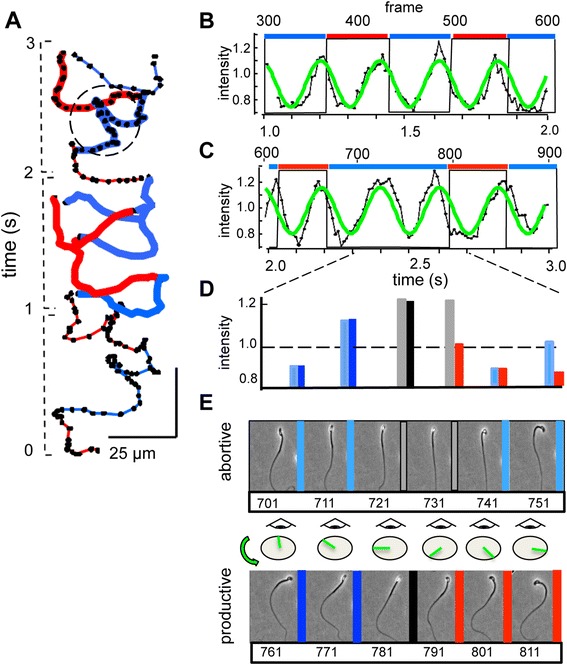
**Both productive and non-productive rolling generate optical signals. (A)** Tracking the centroid of the head of a resting sperm in Ca^2+^-deficient medium HS0Ca. Heavy lines indicate orientation during the t = 1 to 2 s (frames 300 to 600) and 2 to 3 s (frames 600 to 900) segments that are analyzed in panels B and C. **(B, C)** Sinusoidal variation of intensity of centroid region-of-interest (ROI; green) regularly peaks near transitions between right cheek (RCh) and left cheek (LCh) orientation. In C, the centroid intensity peak at approximately t = 2.4 s (around frame 720) occurs without a transition in orientation. **(D)** ROI intensities during the indicated segment (frames 701 to 811, dashed lines in C, circled in panel A). The left bar of each pair is from frames 701 to 751, the right bar from frames 761 to 811. Blue, red, and gray indicate LCh, RCh, and indeterminate cell orientations. **(E)** Stop-motion images of the same cell at the indicated frames, occurring without (abortive, above) or with (productive, below) changes in cell orientation. The cartoon indicates how an observer facing the oncoming sperm would see the plane of the head roll in a counter-clockwise direction as indicated by the green hour-hand.

Figure 3A tracks 3 s of the swimming path of a representative ‘resting’ sperm examined in Ca^2+^-deficient medium HS0Ca, intended to prevent any activation by HCO_3_^−^ (possibly derived from atmospheric CO_2_) which is known to require external Ca^2+^ [10]. Like the cell activated by added NaHCO_3_ in Figure 1A-G, and the ‘hyperactivated’ cell of Figure 2A-D, the resting cell of Figure 3A had a linear averaged trajectory with alternating episodes of LCh and RCh orientations, each with associated sub-segments of forward and retrograde progression.

Figure 3B shows that during the 1 to 2 s segment of this series (frames 300 to 600), oscillating changes in the intensity of reflected light peaked at, or slightly before, each change in orientation. Similar analysis of the 2 to 3 s segment of this series (frames 600 to 900) in Figure 3C also found oscillating intensities of reflected light. The frequencies of the oscillations for the two segments were indistinguishable, indicating that flagellar beat frequency was unaltered. However, the central portion of the segment (around 2.3 to 2.5 s; frames 700 to 750) contained a complete cycle of intensity oscillation while the cell maintained a LCh orientation. Figure 3D shows that similar variations in intensity occurred during this cycle and in the following cycle (frames 760 to 810), which terminated in a reorientation from LCh to RCh. Images at 33 ms intervals from the two cycles (Figure 3E) clearly show that in the LCh-only cycle (frames 700 to 750) the plane of the cell transiently brightens as its plane reorients vertically, then relapses to the LCh orientation. We note that such abortive reorientation produces a characteristic ‘blip’ (circled in Figure 3A) in the swimming track as the plane of the flagellar wave rolls toward the vertical plane then relaxes. We have observed that such blips in the path trajectories correlate with abortive rolls for at least three cells examined in each of the three media used in Figures 1 and 2.

In contrast to the abortive cycle of frames 700 to 750, in the subsequent ‘productive’ cycle (frames 760 to 810) the cell reorients vertically and brightens, then completes the transition to RCh orientation. The cartoon above the images from this cycle (Figure 3E, bottom panel) shows how an observer facing the oncoming sperm would see the plane of the head rotating in a counterclockwise (CCW) direction. Subjective evaluation of image sequences like those in Figure 3E indicates that for productive rolling the direction of rotation is always CCW.

We conclude that the transition between LCh and RCh orientations is accomplished by rolling around the long axis of the cell. The frequency of rolling is synchronized with the flagellar beat. Each beat cycle produces an attempted roll and an optical signal. However, because some attempted rolls are unsuccessful, the optical signal is not a reliable indicator of rolling.

### Orientation directs circular swimming and tethered spinning for sperm that do not roll

**Figure 4:**
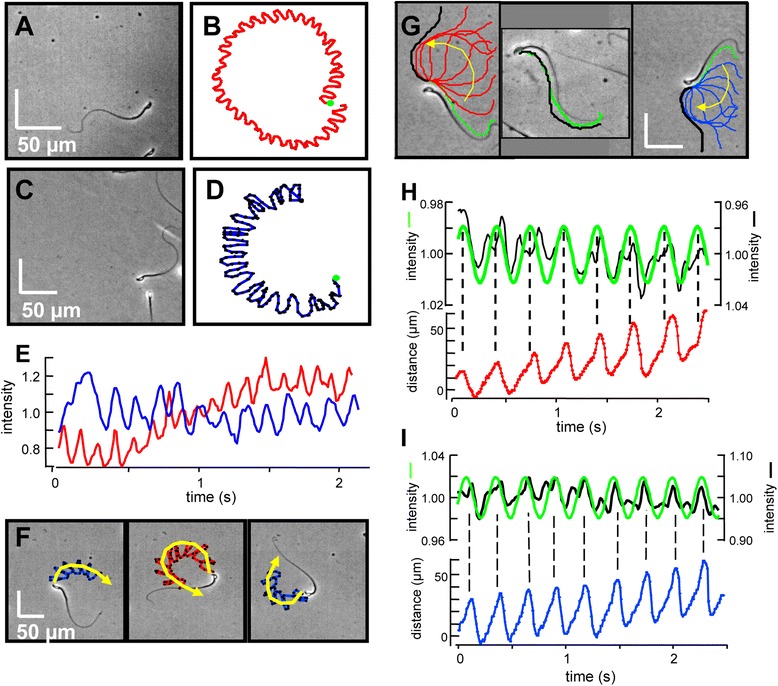
**Orientation directs circular swimming of rolling sperm and tethered spinning for sperm that do not roll. (A, B)** Right cheek (RCh) orientation maintained during a counterclockwise (CCW) circular swimming track. Duration 4.4 s, medium HSB, green dot marks starting point. Scale bar 50 μm. **(C, D)** Left cheek (LCh) orientation and associated clockwise (CW) swimming track. Duration 3 s, medium HSB0Ca. **(E)** Oscillation of centroid region-of-interest (ROI) intensities reports abortive rolling during the circular swimming of cells in A, B (red) and C, D (blue). Rising baseline for cell A results from uneven illumination. **(F)** Sparse rolling abruptly changes swimming trajectories. Tracking of a cell initially in LCh orientation that transiently assumes RCh orientation. Yellow arrows indicate CW and CCW trajectories. Duration 3 s, medium HSB0Ca, scale bar 50 μm. **(G)** RCh (left panel) or LCh (right panel) orientation also determines direction of spinning of tethered sperm in medium HSB0Ca. Central panel shows a tethered sperm (in medium HSBE) that does not spin. Overlaid flagellar traces are offset slightly. Initial (green) and final (black) traces. **(H, I)** Time courses of ROI centroid intensities (black) and best-fit sine functions (green) in upper panels align with flagellar displacements during each beat cycle (lower panels). Data from tethered, spinning cells in left and right subpanels of panel G.

Some sperm swim for extended periods without rolling. Figure 4A,B shows a free-swimming sperm that maintains a RCh orientation. Its swimming path (duration 4.4 s) is circular, with a CCW trajectory. Figure 4C,D shows a different free-swimming sperm that maintains a LCh orientation while swimming a circular path with a clockwise (CW) trajectory. These correlations between orientation and trajectory were observed without exception in >50 cells. Both cells of Figure 4A-D show regularly periodic oscillations in the intensity of reflected light (Figure 4E) resulting from an abortive roll, once per beat cycle.

Still other sperm roll infrequently. Figure 4F shows a cell that completes several beat cycles while swimming a circular CW trajectory in the LCh orientation, then abruptly rolls to the RCh and changes to a CCW trajectory. After completing several more beat cycles, it rolls again to the LCh and resumes a CW circular trajectory. Because no external stimuli were applied, infrequent rolling apparently is controlled by the sperm itself. However, we cannot exclude the possibility that infrequent rolling may be produced by undetected irregularities in the surface of the chambers.

The BSA-containing media used in these studies reduce adherence of sperm to the chamber surface. However, we still often observed sperm that were loosely attached (tethered) to the surface at the base of the head. Attachment prevented tethered sperm from rolling around their long axis to change between LCh and RCh orientation, but many tethered sperm rotated around the attachment site. We term this ‘spinning’, to distinguish it from rolling. The middle panel of Figure 4G shows an image and flagellar traces of a sperm that did not spin. The left panel shows an image and flagellar traces of another cell that began in RCh orientation and completed several beat cycles while spinning in a CCW direction. The cell then abruptly changed to LCh orientation and completed several more beat cycles while spinning in a CW direction (right panel). Additional file 2 shows this example of the linked changes in orientation and the direction of spinning, and of the apparent brief release from attachment that allows the cell to roll from RCh to LCh.

Figure 4H shows that the sperm tethered in RCh orientation in the left panel of Figure 4G still produced small (± 1 to 2%) oscillations in reflected light intensity (upper panel). These oscillations were synchronized with the flagellar beat as reported by cyclical displacement of the flagellum along a linescan perpendicular to the axis of the flagellar beat (lower panel). Figure 4I shows that this synchronization also was maintained when the cell changed to LCh orientation (right panel of Figure 4G). Despite the abrupt change in cell orientation, neither the time course of the oscillating optical signal nor the flagellar beat frequency was altered.

We conclude that RCh or LCh orientation determines the bias of the asymmetrical flagellar waveform and therefore both the trajectory of free-swimming sperm and the direction of spinning of tethered sperm. Infrequent, spontaneous rolling produces abrupt changes in path trajectories of free-swimming sperm and in the direction of spinning of tethered sperm.

### Release from transient attachment allows changes in orientation and new trajectories

**Figure 5:**
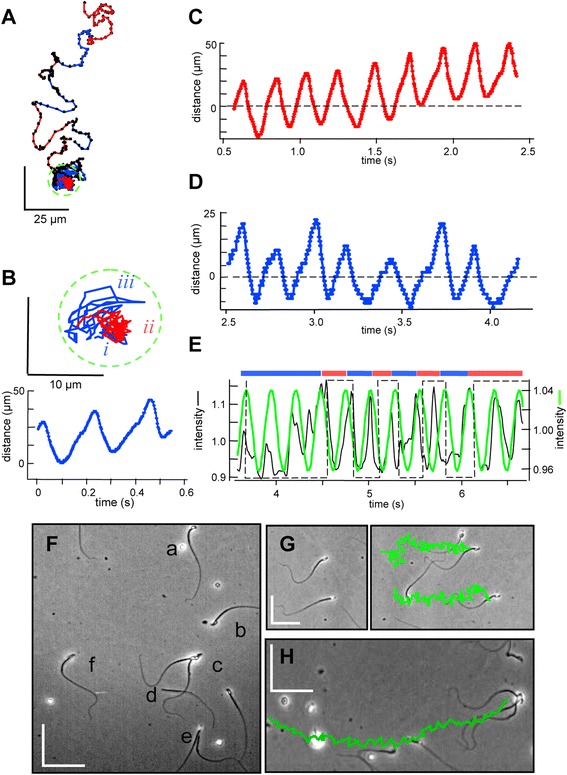
**Release from transient attachment allows change in orientation and trajectory. (A)** Tracking a transiently attached sperm. Duration 6 s, sampling 100 frames per second (fps), medium HSB. Right cheek (RCh, red) and left cheek (LCh, blue) orientations are indicated. Green circle encloses initial 4 s period of attachment. **(B-D)** Time courses of changes in flagellar location (from linescans perpendicular to the flagellar axis at approximately 15 μm from the flagellar origin) during attachment. (**B)** Inset shows expanded view of track during attachment, divided into segments of LCh (*i*, *iii*) and RCh (*ii*) orientation. Time course for segment *i*. **(C, D)** Time courses for subsequent RCh (*ii*) and LCh (*iii*) attachment. For C and D, images were first ‘despun’ by rotation of 0.1 degrees per frame. **(E)** Time courses of centroid region-of-interest intensities (black) and best-fit sine functions (green) during subsequent free-swimming of the released sperm. Bars indicate periods of LCh (blue) and RCh (red) orientation. **(F)** Coexistence of various swimming behaviors in a single 240 × 350 μm field. Medium HSB. Lower case letters indicate cells showing: (a, b) linear swimming by two unattached sperm - sperm **a1** exits at t = 0.2 s, sperm **a2** enters at t = 3.5 s; (c) tethered spinning of an attached sperm duo; (d) linear swim by an unattached, headless sperm; (e) transient linear swim terminated by attachment at t = 3.5 s; (f) transient tether followed by linear free swim (the cell examined in A-E). **(G, H)** Tracking of cells from other fields of this same preparation, examined under the same conditions as in panel A except for use of medium HS rather than HSB. **(G)** Two cells at the start (left) and end (right) of a 2 s linear swim. **(H)** A trio of attached cells at the end of a 2 s linear swim. Swimming tracks are green. Scale bars, 50 μm.

Together, Figures 1, 2, 3 and 4 show that spontaneous changes in RCh and LCh orientation produce abrupt changes in the behavior of free-swimming and tethered sperm. Figure 5A tracks a loosely tethered sperm that spontaneously detached to swim freely. Figure 5B (upper panel) shows that the initial approximately 4 s of tethered attachment had two segments of LCh orientation (*i* and *iii*) with an intermediate RCh segment (*ii*). Linescans perpendicular to the beat axis showed periodic displacement of the flagellum during the first LCh segment (lower panel) indicating a beat frequency of 3.9 ± 0.1 Hz. Linescans applied to the subsequent RCh and LCh segments (Figure 5C,D) reported little or no change in beat frequency. The beat frequency indirectly reported by the oscillating optical signal (Figure 5E) also did not change when the cell detached at t around 4.2 s to swim freely with periodic rolling.

We conclude that attachment of sperm to surfaces can be reversible, allowing either reorientation then reattachment (as in Figures 4G-I and 5B-D, and Additional file 2), or a transition to free-swimming trajectories (Figure 5E) with periodic rolling and reorientation.

### Multimodal attachments increase diversity in swimming

In these experiments, single fields of view (approximately 400 × 400 μm, or parts thereof) often contained sperm demonstrating a variety of behaviors. Figure 5F shows an initial frame from a 6 s duration sequence of the same field that imaged the cell of Figure 5A-E. In addition to that transiently attached cell (f), we also observed three different free-swimming sperm that entered and/or left the field (a,b); a tethered ‘duo’ - two cells attached together at the head (c); a headless free-swimming sperm (d); and a transiently free-swimming cell (e) that subsequently became surface-tethered. The video movie of this sequence (Additional file 3) also shows several immotile cells, and demonstrates how the distribution of swimming behaviors changes moment to moment, preventing us from making quantitative statements about the relative abundance of various sperm swimming behaviors.

Single fields of view also often allowed comparison of the swimming paths and speed of two or more cells that pursued similar trajectories. Figure 5G shows the starting (left) and ending (right) frames from such a ‘race’ between two single, free-swimming cells. Their approximately linear, mean trajectories had similar path speeds (194 ± 12 versus 187 ± 6 μm s^−1^) and rates of progression (56 ± 4 versus 55 ± 4 μm s^−1^). Visual examination confirmed that both cells rolled at regular intervals to produce alternating lateral excursions from the mean path.

In another field (Figure 5H), we tracked the trajectory of a group of three non-rolling sperm, attached to each other at the head. The swimming path of this ‘trio’ also was approximately linear. Although their path speed (175 ± 11 μm s^−1^) was similar to that of the single sperm of Figure 5G, their rate of progression (106 ± 2 μm s^−1^) was much faster, suggesting that attachment to other sperm can produce cooperative swimming behavior.

## Discussion

### Capture and release of sperm from an oviductal reservoir

In early work, *ex vivo* examinations of the oviducts of mated mice revealed a putative sperm reservoir, apparently formed by binding of the sperm head to epithelial cells lining the lower portion of the oviduct. Because only hyperactivated sperm were seen to detach from the epithelium, it was proposed that a spontaneous onset of hyperactivation controls release of sperm from this reservoir [20,21]. This conjecture is consistent with calculations that hyperactivation may produce increased torsional forces [22], perhaps sufficient to cause release. However, other *ex vivo* observations prompted a contrasting view of the sperm reservoir. Specifically, periovulatory release of sperm from the porcine oviductal epithelium suggested that the ovary produced the signal(s) for release [23]. For human sperm this proposed signal might be progesterone, which promotes opening of the CatSper cation channel [24,25] to allow the entry of Ca^2+^ that is required for hyperactivation [6,9,11,26,27]. Thus, at least for humans, progesterone released at ovulation might signal increased hyperactivation and detachment of sperm.

### What is the significance of diverse swimming behaviors?

In past *in vitro* studies we examined individual, tethered epididymal mouse sperm [6–13,15] and ejaculated human sperm [15] in simple media at room temperature. We have now extended this approach to include monitoring of the behavior of free-swimming, unattached sperm. The results reveal surprising diversity in the type and duration of attachments of sperm to each other and to the chamber surface. Because attachments are formed and terminated in the absence of applied stimuli, they apparently reflect alterations at the sperm surface, presumably controlled by unknown intracellular processes. If these alterations also evoke detachment of sperm from epithelial cells *in vivo*, then the basis for at least some portion of release from the sperm reservoir may be unrelated to hyperactivation.

*In vitro*, mouse sperm attach to the chamber surface at the apical hook, or more commonly, at a site in the posterior head near the equatorial segment. We found that attachment to other sperm also usually occurred at the head and involved the apical hook. Thus, it is likely that the same adherent sites are used for attachments to surfaces or to other cells. Several groups [28,29] have reported sperm-to-sperm attachments for various other murid rodent species [30]; seen effects on swimming behavior, including increased progression [29]; and speculated about possible cooperative roles in sperm competition in polyandrous matings [31,32], or in the evolution of genes expressed post-meiotically in sperm [33]. We propose that sperm-sperm attachments *in vivo* also might allow sperm to avoid, or terminate, attachment in the oviductal reservoir or elsewhere in the female reproductive tract.

### Why do sperm roll?

In contrast to these speculations about the processes that occur *in vivo* and their significance, we can draw several firm conclusions about the basis of several aspects of sperm swimming behavior *in vitro*. Most past studies of mammalian sperm swimming behavior used video records obtained with exposure times too long to produce clear images of the flagellum. Therefore, reported data was derived from tracking changes in the location of the sperm head [16,17,34]. Here we have used shorter (approximately 2 ms) exposures to obtain sharply focused, stop-motion images of both head and flagellum. Importantly, because the head of mouse sperm has a falciform morphology, we could simultaneously follow changes in the location and in the orientation of the plane of head (its ‘sidedness’). Several strong correlations became apparent.

First, we found that sperm with linear mean swimming paths showed regularly periodic changes in sidedness, which balance the opposing biases in path trajectories that result from the asymmetry of the flagellar beat. Although the mean trajectory was linear, the associated swimming path staggers. Such staggering lateral excursions also are apparent in published records of linear mean swimming paths of human sperm [16,17], and presumably have a similar previously unrecognized basis, obscured by the symmetrical spatulate morphology of the head of human (and most other mammalian) sperm.

Conversely, mouse sperm with circular swimming paths did not undergo changes in sidedness. Because sidedness determined the direction of bias of the flagellar beat, it also determined whether the cell circled in a CW or CCW direction. These results indicate that the extent of asymmetry of the flagellar beat is the probable primary determinant of the radius of the circular trajectories of free-swimming sperm. This prediction is consistent with the finding that path curvature of bull sperm is directly related to averaged flagellar curvature [35]. It also may be consistent with modeling studies [36] that show that circular trajectories are determined by the chiral asymmetry of the sperm shape. The same associations between sidedness and the direction of rotation around the attachment point (spinning) are found in tethered sperm. Spinning presumably is maintained by the asymmetry of the flagellar beat.

Second, we saw that the changes in sidedness were produced by the rolling of sperm around its long axis. Our results indicate that each flagellar beat cycle generates torque that produces either a productive or an abortive roll. The direction of productive rolling appeared to be always CCW, in agreement with another recent report [2] and with early findings for hamster sperm [37]. When an attempted roll aborted, the tilt of plane of the head relaxed by CW rotation. Past reports of CW rolling [38] may have been observations of such relaxation from attempted rolls. Assessment of the direction of rolling by a more objective and quantifiable approach remains a desirable goal for future work.

Other past studies disagree on the significance of sperm rolling and on its prevalence [2,37,39,40]. Recognition of the three-dimensional nature of swimming trajectories [41] and advanced holographic imaging techniques have produced recent discovery of their chiral ribbon nature [42]. We speculate that productive rolling produces helical ribbon swimming paths and that abortive rolling produces twisted ribbon paths. Thus, we propose that the significance of rolling is that it is a primary determinant of sperm swimming behavior, as demonstrated by the linkages of rolling to changes in sidedness and to path trajectories.

The prevalence of rolling is difficult to quantify. If we accept that each beat cycle generates torque for an attempted or a productive roll, then the question becomes: What limits or promotes productive rolling? When we examined sperm in media that produce resting, activated, or hyperactivated motility, no noticeable differences were seen in the distribution between productive and abortive rolling. Other possible influencing factors, such as the depth of the incubation chamber, exposure to seminal components, or viscosity of the medium will require further, systematic investigation. Past work [43–45] reports that sperm become ‘trapped’ when swimming in the proximity of surfaces. Here, the use of 20 μm deep chambers presumably promotes such near-surface swimming. It remains unclear whether such trapping prevents sperm rolling and thus promotes circular swimming trajectories.

For both productive and abortive rolling, tilting of the plane of the head changed its reflectivity and produced a characteristic optical signal. Smaller, but still detectable, signals were produced even by tethered sperm. Apparently the torque that caused rolling of unattached sperm also caused a slight tilt of tethered sperm, which then relaxed without breaking the attachment to the chamber surface.

Third, we observed that transitions from abortive to productive rolling produced abrupt changes in trajectory. Sperm swimming in circular paths occasionally underwent a single productive roll and an immediate change from a CW to CCW direction (or vice versa). Similarly a few tethered sperm spontaneously detached to swim freely, sometimes then reattaching after one or more episodes of productive rolling. If reattachment produced a change in sidedness, then it was accompanied by a change in the direction of spinning about the attachment site.

### Rolling, hyperactivation, rheotaxis, and attachments

Miki and Clapham [2] report that rates of rolling (as reported by the frequency of oscillations in intensity of reflected light) increased more than two-fold for mouse sperm incubated under conditions intended to produce hyperactivation. By contrast, we found that the rate of rolling (also reported by changes in light intensity) was slower for hyperactivated sperm and remains correlated with the slower flagellar beat frequency seen here and reported in prior work [11].

Miki and Clapham [2] conclude that rheotaxis is a consequence of rolling, and that rolling is actively controlled by CatSper-mediated entry of Ca^2+^. We have not examined behavior of free-swimming CatSper-null mutant sperm, nor monitored sperm rheotaxis. However, we found that the rate of rolling of wild-type sperm decreased only slightly and remained linked to the flagellar beat frequency for sperm incubated in media without added Ca^2+^, with or without added ethylene glycol tetraacetic acid (EGTA). Hence for sperm examined in the absence of rheotactic flow, entry of external Ca^2+^ does not modulate the linkage between rolling and the flagellar beat. It remains possible that flow engages such a modulatory mechanism.

Although sperm-to-sperm attachments can increase progression *in vitro*, such attachments also prevent rolling. However, if rolling is required for rheotaxis, then sperm aggregation might diminish progression in the oviduct *in vivo*. Aggregation might still promote selection by operating transiently, for instance to increase progress through the utero-tubal junction.

## Conclusions

We found that mouse sperm have an unexpectedly broad repertoire of swimming behaviors. Moreover, sperm can rapidly change between different swimming modes to produce abrupt changes in trajectory, suggesting a means for possible tactic or avoidance responses to external cues. The ability of sperm to form and break attachments to surfaces or to other cells suggests additional possibilities for avoiding or overcoming barriers to their progress through the female reproductive tract. At a more mechanistic level, we found that the trajectories of free-swimming sperm were determined largely by the presence or absence of rolling of the sperm around their long axis. Although it remains unclear what determines whether sperm roll or not, we did find that rolling was not affected by manipulations known to engage or preclude engagement of the cAMP- and Ca^2+^-mediated pathways that control the frequency and asymmetry of the flagellar beat.

## Methods

### Sperm preparation

After isoflurane anesthesia and cervical dislocation, sperm were released from excised cauda epididymides and vasa deferentia of C57BL/6J mice as described previously [13]. Sperm were allowed to swim out in HS buffer (in mM: 135 NaCl, 5 KCl, 2 CaCl_2_, 1 MgCl_2_, 20 HEPES, 5 glucose, 10 DL-lactic acid, and 10 pyruvic acid, pH 7.4) for 20 min at 37°C and 5% CO_2_. After two washing steps (3 min at 400 × g), sedimented sperm were resuspended to a final concentration of 1 to 2 × 10^7^ cells/ml in the following buffers, each supplemented with 5 mg/ml BSA and adjusted to pH 7.4. The medium for resting sperm was buffer HS, or for activated sperm buffer HSB (buffer HS with 15 mM NaHCO_3_). Hyperactivated sperm were examined after incubation in HSB buffer for 150 min at 37°C with 5% CO_2_. For the Ca^2+^-deficient experiments, sperm were examined in HS0Ca or HSB0Ca buffers (buffer HS or HSB prepared without added CaCl_2_). Some experiments used HSBE (buffer HSB prepared without added CaCl_2_ and containing 1 mM EGTA).

### Image collection and analysis

High-speed images were collected immediately after 10 μl of sperm suspension were transferred into a 20 μm deep Cell-Vu DRM-600 Chamber (Advanced Meditech Int., Little Neck, NY, USA) or into a 10 μm deep Makler Counting Chamber (Medical Instruments, Herford, Germany). Chambers were examined on an upright phase contrast microscope (Olympus CX41) using a PlanCN 10X phase 1 0.25 numerical aperture objective and equipped with an IDT M3 high-speed camera (12 × 12 μm pixels; IDT Inc., Tallahassee, FL, USA). Images were recorded at 300 frames per second for a total time of 6.6 s. Flagellar waveform analysis was performed as previously described [7,11,12]. Briefly, determinations of flagellar beat frequency and shear analysis used software routines written in the Igor-Pro™ (Wavemetrics, Lake Oswego, OR, USA) programming environment. Swimming tracks were extracted with the MTrack2 ImageJ plugin [46]. Various other ImageJ plug-ins allowed analysis of optical signals.

### Statistics

Averaged results are presented as mean ± standard error of the mean.

## Additional files

Additional file 1:
**Linear trajectory of the rolling sperm of Figure** 1 **A-E.** Duration 2 s. Sampling 150 fps. Bar, 2 μm. Additional file 2:
**Transitional circular trajectories of the tethered sperm of Figure** 4 **G.** Duration 6.6 s. Sampling 100 fps. Orientation changes from LCh to RCh and trajectory changes from CW to CCW at 2.78 s. Additional file 3:
**Coexistence of multiple swimming modes in the single field of view of Figure** 5 **F.** Duration 4.0 s. Sampling 50 fps.

## Abbreviations

BSA: bovine serum albumin
CCW: counterclockwise
CW: clockwise
Fps: frames per second
HS: HEPES-saline
HS0Ca: HEPES-saline without added Ca2^+^
HSB: HEPES-saline-bicarbonate
HSE: HEPES-saline-EGTA
LCh: left cheek
RCh: right cheek
